# Co-occurrence of Autism Spectrum Disorder and Gender Incongruence in an Adolescent Female Patient: Diagnostic Challenges in an Underrecognized Clinical Association

**DOI:** 10.7759/cureus.112782

**Published:** 2026-07-16

**Authors:** Christian David Galindo, Angelica Maria Perez Camacho, Ramon Eduardo Lopera Lopera, Natalia Cardona Lopez, Stefanía Villada Arenas

**Affiliations:** 1 Psychiatry, Universidad Internacional Valencia, Valencia, ESP; 2 Psychiatry, Corporación Universitaria Remington, Medellín, COL; 3 Psychiatry and Behavioral Sciences, Mental Antioquia Hospital, Medellín, COL; 4 Child and Adolescent Psychiatry, Clínica Servid, El Carmen de Viboral, COL; 5 Psychiatry and Behavioral Sciences, Clínica Servid, El Carmen de Viboral, COL; 6 Pediatrics, Corporación Universitaria Remington, Medellín, COL

**Keywords:** autism spectrum disorder (asd), clinical case report, gender-diversity, gender incongruence, neurodevelopmental disorders

## Abstract

Autism spectrum disorder (ASD) and gender incongruence (GI) are increasingly recognized as co-occurring conditions, with growing evidence indicating a prevalence substantially higher than expected in the general population. This association presents important diagnostic challenges, particularly among adolescents assigned female at birth (AFAB), in whom ASD frequently remains unrecognized because of the female autism phenotype, social camouflaging strategies, and overlapping psychiatric manifestations. Delayed identification of ASD may contribute to significant psychological distress and increased psychiatric morbidity.

We report the case of a 16-year-old adolescent AFAB with persistent gender incongruence who was admitted to a child and adolescent psychiatric unit because of severe depressive symptoms, recurrent self-injurious behavior, chronic suicidal ideation, and multiple suicide attempts. Retrospective developmental assessment revealed longstanding difficulties in social communication and reciprocity, sensory hypersensitivities, cognitive rigidity, restricted interests, and marked interpersonal difficulties dating back to early childhood. A comprehensive psychiatric, neuropsychological, and developmental evaluation, including administration of the Autism Diagnostic Interview-Revised (ADI-R), supported a diagnosis of ASD consistent with the female autism phenotype.

This case highlights the diagnostic complexity associated with the coexistence of ASD and GI and underscores the importance of comprehensive neurodevelopmental assessment in gender-diverse adolescents presenting with severe psychiatric symptoms. Early recognition of ASD may improve diagnostic accuracy, facilitate individualized treatment planning, and support better psychiatric and psychosocial outcomes in this particularly vulnerable population.

## Introduction

The relationship between autism spectrum disorder (ASD) and gender incongruence (GI) has emerged as a topic of growing interest within child and adolescent psychiatry, reflecting an increasing recognition of the complex interplay between neurodevelopment, identity formation, and mental health. ASD is a neurodevelopmental condition characterized by persistent deficits in social communication and interaction, accompanied by restricted and repetitive patterns of behavior, interests, or activities [1]. Affecting approximately 1%-2% of the global population, ASD is associated with a substantial burden of psychiatric comorbidity, including anxiety disorders, depression, self-injurious behaviors, and suicidality [2]. These difficulties frequently intensify during adolescence, a critical developmental period marked by rapid psychosocial changes, identity consolidation, and increased vulnerability to mental health challenges.

GI, as defined by the International Classification of Diseases, Eleventh Revision (ICD-11), refers to a marked and persistent incongruence between an individual's experienced gender and assigned sex at birth [3]. Although gender diversity is recognized as a natural variation of human experience, adolescents experiencing GI often face significant psychosocial stressors, including stigma, discrimination, family conflict, and social exclusion. Consequently, they are at increased risk of depression, anxiety, self-harm, and suicidal behavior. The coexistence of GI and ASD therefore represents a particularly complex clinical scenario, given that both conditions independently confer heightened vulnerability to emotional distress and functional impairment [4].

Over the past decade, a growing body of evidence has consistently demonstrated a significant association between ASD and GI. Gender variance has been reported in approximately 3.8%-7.2% of individuals with ASD, whereas autistic traits or formal ASD diagnoses have been identified in 5.5%-21.3% of gender-diverse populations, rates substantially exceeding those observed in the general population [5]. Furthermore, studies conducted in specialized gender identity clinics have identified an overrepresentation of ASD diagnoses among children and adolescents referred for gender-related concerns. This relationship appears to be bidirectional, suggesting that autistic individuals are more likely to experience gender diversity, while gender-diverse youth exhibit higher rates of autistic characteristics than expected in the general population. Notably, this overlap has been reported more frequently among individuals assigned female at birth (AFAB), a finding that has attracted increasing attention in light of the well-documented underrecognition of ASD in girls and young women.

Despite the consistency of these epidemiological findings, the mechanisms underlying the association between ASD and GI remain incompletely understood. Several explanatory models have been proposed, encompassing neurobiological, cognitive, developmental, and sociocultural perspectives. These include prenatal hormonal influences, differences in social cognition and identity development, cognitive rigidity, reduced conformity to social expectations, and distinct patterns of self-perception and social information processing. However, none of these hypotheses has been consistently supported across studies, underscoring the heterogeneity and complexity of this clinical association. Current evidence increasingly favors a multifactorial framework in which biological predispositions interact dynamically with psychological, developmental, and environmental influences throughout the process of gender identity formation [6].

From a clinical perspective, the coexistence of ASD and GI presents substantial diagnostic and therapeutic challenges. Difficulties in social communication, emotional insight, cognitive flexibility, executive functioning, and self-awareness may influence how adolescents explore, interpret, and express gender-related experiences. Consequently, distinguishing persistent GI from gender-related concerns that may be influenced by restricted interests, cognitive rigidity, or other manifestations of ASD can be particularly challenging. These complexities are further compounded by the need to balance timely access to gender-affirming care with careful diagnostic assessment. For this reason, current clinical guidelines emphasize comprehensive, longitudinal, and multidisciplinary evaluations that integrate developmental, psychiatric, psychological, and family perspectives to support individualized and evidence-based decision-making [7].

These challenges are particularly relevant among adolescent females, a population in which ASD frequently remains underrecognized because of subtler clinical presentations, compensatory social camouflaging strategies, and phenotypic characteristics that differ from traditional male-centered diagnostic models [8]. As a consequence, many girls remain undiagnosed for years, delaying access to appropriate interventions and increasing the risk of emotional distress, social isolation, and psychiatric comorbidity. This diagnostic gap may be especially significant among gender-diverse adolescents, in whom overlapping developmental, psychological, and social factors can further obscure recognition of underlying neurodevelopmental difficulties [9].

Although awareness of the overlap between ASD and GI has increased substantially in recent years, important gaps remain regarding its clinical presentation, diagnostic assessment, and management in female adolescents. Improved recognition of this intersection is essential to facilitate timely diagnosis, optimize treatment planning, and promote better psychosocial outcomes. In this context, we present the case of an adolescent female patient with persistent GI whose comprehensive psychiatric and neurodevelopmental evaluation led to the diagnosis of previously unrecognized ASD. This case highlights the diagnostic complexities associated with this increasingly recognized yet still insufficiently understood clinical association and underscores the need for developmentally informed, multidisciplinary approaches capable of addressing the unique needs of gender-diverse autistic youth.

## Case presentation

A 16-year-old adolescent AFAB, who consistently identified as a transgender male since early adolescence, was admitted to a specialized child and adolescent psychiatric inpatient unit due to a severe depressive episode characterized by persistent suicidal ideation, multiple previous suicide attempts, recurrent self-injurious behavior, and high suicide risk. At the time of admission, the patient was attending 10th grade and lived with maternal grandparents and a younger sibling. Family history was notable for depression in a second-degree relative and cerebrovascular disease in the maternal grandfather.

A retrospective reconstruction of the developmental history revealed longstanding neurodevelopmental and socioemotional difficulties predating both the onset of affective symptoms and gender-related concerns. Pregnancy was considered high-risk because of threatened miscarriage and multiple maternal hospitalizations, culminating in a preterm cesarean delivery due to failure to progress in labor. Early developmental milestones were delayed, with independent walking and the emergence of meaningful speech occurring at approximately two years of age. During childhood, the patient also required follow-up for motor coordination difficulties and developmental delays.

From an early age, caregivers reported persistent difficulties in social communication and socioemotional reciprocity. The patient was described as emotionally reserved, with limited social initiative, reduced emotional expressiveness, selective interpersonal relationships, and a strong preference for solitary activities. Social interactions often depended on the initiative of others, while understanding implicit social rules and adapting to complex interpersonal situations appeared particularly challenging. Retrospectively, the patient reported a persistent sense of being different from peers and a need to consciously modify behavior in order to achieve social acceptance.

Several behavioral characteristics suggestive of ASD were identified throughout development. These included marked cognitive rigidity, a strong need for predictability, adherence to routines and rules, significant distress in response to unexpected changes, and highly restricted interests of unusual intensity. During childhood and adolescence, the patient developed intense interests in computers, the English language, and foreign cultures, devoting substantial amounts of time to these activities. Repetitive organizational behaviors, categorization rituals, and a prolonged fascination with moving objects and rotating visual stimuli were also reported.

Sensory processing abnormalities were a prominent feature throughout development. The patient exhibited hypersensitivity to auditory, visual, and tactile stimuli, including intolerance to repetitive or high-pitched sounds, discomfort with specific clothing textures, sensitivity to bright lights, and food selectivity largely driven by sensory characteristics. Repetitive self-regulatory behaviors, particularly body rocking during periods of stress, anxiety, or sensory overload, were also observed.

Social and adaptive difficulties became increasingly evident during the school years. Despite initially demonstrating adequate academic performance, the patient experienced progressive functional decline associated with persistent interpersonal difficulties, bullying, social isolation, and increasing reliance on online interactions as the primary source of social engagement. Interpersonal relationships were characterized by emotional dependency, difficulties establishing boundaries, and a strong need for social acceptance, all of which contributed significantly to psychological distress.

Gender-related concerns emerged at approximately 12 years of age, initially within online communities and later extending to other social contexts. From that time onward, the patient consistently identified with a male gender identity, reporting greater comfort when perceived socially as male and expressing a persistent desire to be recognized accordingly. This identification remained stable over time and appeared independent of fluctuations in mood symptoms.

Concurrently, the patient developed progressively severe psychopathology characterized by persistent depressed mood, anhedonia, feelings of worthlessness, hopelessness, social withdrawal, sleep disturbances, reduced appetite, and recurrent thoughts of death. Self-injurious behavior through cutting began several years prior to hospitalization and later progressed to multiple suicide attempts involving both self-inflicted injuries and medication overdoses. At admission, persistent suicidal ideation with structured planning, marked impulsivity, poor insight, and profound hopelessness were documented.

Mental status examination revealed markedly reduced eye contact, monotonous prosody, limited affective modulation, restricted emotional reciprocity, and predominantly concrete thinking. Speech was coherent but minimally spontaneous. Affect was depressed, constricted, and characterized by low emotional resonance. No psychotic symptoms, perceptual disturbances, manic features, or formal thought disorder were identified. Insight was limited, and the patient demonstrated significant difficulties recognizing and communicating internal emotional states.

During hospitalization, treatment was initiated with sertraline 25 mg/day and subsequently titrated to 50 mg/day, risperidone 1 mg/night, pregabalin 50 mg three times daily, and trazodone 25-50 mg/night for insomnia. Pharmacological management was complemented by individual psychotherapy, family interventions, group therapy, and suicide prevention strategies aimed at emotional stabilization and diagnostic clarification.

Given the coexistence of persistent social communication difficulties, sensory abnormalities, cognitive rigidity, restricted interests, social isolation, and gender incongruence, a comprehensive neuropsychological assessment was conducted. Cognitive evaluation using the Wechsler Intelligence Scale for Children-Fifth Edition (WISC-V) revealed a Full-Scale Intelligence Quotient (FSIQ) of 87, with a heterogeneous cognitive profile characterized by relative strengths in working memory, visuospatial reasoning, and fluid reasoning, alongside significantly lower performance in verbal comprehension and processing speed.

The diagnostic assessment was further supplemented by administration of the Autism Diagnostic Interview-Revised (ADI-R), which revealed clinically significant impairments across the domains of reciprocal social interaction, communication, and restricted and repetitive patterns of behavior. Behavioral observations during assessment documented reduced eye contact, atypical prosody, limited facial expressiveness, impaired socioemotional reciprocity, rigid adherence to routines, and persistent social communication difficulties. Importantly, the patient reported a longstanding need to consciously monitor and modify behavior in social situations to facilitate peer acceptance, a pattern consistent with social camouflaging frequently described in autistic females and recognized as a major contributor to delayed diagnosis.

Integration of developmental history, caregiver reports, psychiatric assessment, neuropsychological findings, ADI-R results, and direct clinical observation supported a diagnosis of Autism Spectrum Disorder according to DSM-5-TR criteria. The final diagnostic formulation included ASD, GI, severe depressive episode without psychotic features, recurrent self-injurious behavior, and suicidal behavior. The late identification of the underlying neurodevelopmental condition provided a comprehensive explanatory framework for the patient's longstanding social difficulties, psychiatric vulnerability, sensory abnormalities, and gender-related experiences. Furthermore, the presence of social camouflaging strategies and a clinical presentation consistent with the female autism phenotype likely contributed to the delayed recognition of ASD throughout adolescence.

## Discussion

This case illustrates the diagnostic complexity associated with the coexistence of ASD and GI in an adolescent AFAB, highlighting the challenges of recognizing autism in individuals who present with the female autism phenotype and significant psychiatric comorbidity [10]. Although the patient had exhibited developmental, behavioral, and sensory features consistent with ASD since early childhood, the diagnosis remained unrecognized until adolescence, when a comprehensive psychiatric and neurodevelopmental assessment was undertaken during hospitalization for severe depression, recurrent self-injurious behavior, and multiple suicide attempts [11]. This diagnostic trajectory underscores the importance of considering underlying neurodevelopmental conditions in gender-diverse adolescents presenting with complex psychiatric symptoms.

Growing evidence supports a clinically meaningful association between ASD and GI. Epidemiological studies have consistently demonstrated higher rates of autistic traits and formal ASD diagnoses among gender-diverse individuals, while gender diversity appears to be disproportionately represented within autistic populations. Although prevalence estimates vary across studies because of methodological heterogeneity and differences in diagnostic approaches, recent systematic reviews and meta-analyses suggest that this overlap is significantly greater than would be expected by chance alone [12]. These findings have contributed to increasing recognition of ASD and GI as conditions that frequently coexist and may interact in clinically relevant ways.

The present case reflects many of the developmental and behavioral characteristics previously reported in individuals with co-occurring ASD and GI. Long before the emergence of gender-related concerns, the patient demonstrated persistent deficits in social communication and reciprocity, sensory hypersensitivities, cognitive rigidity, restricted interests, and marked difficulties in peer relationships. Importantly, GI emerged several years after the onset of these neurodevelopmental features and remained stable over time, supporting its conceptualization as a distinct and enduring aspect of identity development rather than a transient manifestation of autistic restricted interests or cognitive rigidity [13]. This distinction is particularly relevant because one of the principal clinical challenges in this population involves differentiating persistent gender incongruence from gender-related concerns that may be influenced by broader neurodevelopmental characteristics.

A particularly relevant aspect of this case is the delayed recognition of ASD despite the presence of multiple developmental indicators dating back to early childhood. This finding reflects a growing body of evidence suggesting that autistic females frequently remain underrecognized because their clinical presentation differs from the traditional male-centered diagnostic model upon which many screening and diagnostic practices were originally developed [14]. In contrast to the more overt manifestations commonly observed in males, autistic girls often exhibit greater social motivation, subtler communication difficulties, fewer obvious repetitive behaviors, and more sophisticated compensatory strategies. Consequently, developmental difficulties may remain partially concealed until adolescence, when increasing social demands exceed compensatory capacities and psychiatric symptoms become the primary focus of clinical attention. In the present case, social camouflaging emerged as a clinically relevant phenomenon, as the patient described a longstanding need to consciously observe, imitate, and modify social behaviors in order to achieve peer acceptance [15]. This adaptive but cognitively demanding strategy likely contributed to years of unmet neurodevelopmental needs and delayed recognition of ASD despite a developmental history strongly suggestive of an underlying neurodevelopmental condition.

The psychiatric burden observed in this patient highlights the potential consequences of the intersection between ASD and GI. Previous studies have demonstrated that both conditions are independently associated with elevated rates of depression, anxiety, self-harm, and suicidal behavior [16]. However, when they coexist, the resulting psychological burden may be substantially amplified. Recurrent self-injurious behavior, multiple suicide attempts, chronic suicidal ideation, severe depressive symptoms, and profound social isolation were all present in this case and are consistent with reports identifying autistic gender-diverse youth as one of the highest-risk groups for adverse mental health outcomes. Rather than representing two independent clinical conditions, ASD and gender incongruence may create a cumulative vulnerability characterized by heightened exposure to psychosocial stressors and reduced access to protective factors. Several mechanisms have been proposed to explain this increased vulnerability, including minority stress, bullying, social exclusion, impaired social connectedness, emotional dysregulation, stigma-related experiences, and reduced access to developmentally appropriate support systems [17]. The convergence of these factors may contribute to escalating psychological distress and increase vulnerability to suicidal behavior.

The mechanisms underlying the association between ASD and GI remain incompletely understood and are likely multidimensional. Several explanatory frameworks have been proposed, including differences in social cognition, identity development, sensory processing, cognitive flexibility, and reduced adherence to conventional gender norms. Nevertheless, no single model adequately explains the considerable heterogeneity observed across studies. Current evidence increasingly supports a multifactorial perspective in which biological predispositions interact dynamically with developmental experiences, psychological processes, and sociocultural influences throughout identity formation and development [18]. The present case is consistent with this conceptualization, as both neurodevelopmental vulnerabilities and environmental stressors appeared to contribute to the patient's developmental trajectory, gender-related experiences, and psychiatric presentation.

Another important contribution of this case relates to the diagnostic value of comprehensive neurodevelopmental assessment in gender-diverse adolescents presenting with severe psychiatric symptoms. The diagnosis of ASD was not established solely on the basis of current symptom presentation but emerged through the integration of developmental history, caregiver reports, direct clinical observation, neuropsychological evaluation, and administration of the ADI-R. The ADI-R proved particularly valuable because it systematically documented longstanding abnormalities in reciprocal social interaction, communication, and restricted and repetitive patterns of behavior that had not been adequately recognized during previous psychiatric evaluations [19]. This finding reinforces current recommendations emphasizing the importance of structured developmental assessments when autism is suspected in gender-diverse youth and highlights the risk of diagnostic overshadowing when psychiatric symptoms dominate the clinical picture.

From a clinical perspective, this case underscores the importance of routinely considering ASD in gender-diverse adolescents presenting with complex psychiatric symptoms, particularly when developmental histories reveal longstanding difficulties in social reciprocity, sensory processing, cognitive flexibility, or restricted interests. Recognition of co-occurring neurodevelopmental conditions should not be viewed as a barrier to the assessment or management of gender incongruence. Rather, it may facilitate more accurate diagnostic formulation, individualized treatment planning, and the implementation of developmentally informed interventions tailored to the specific needs of autistic gender-diverse youth.

Although causal relationships cannot be inferred from a single case, detailed clinical reports remain valuable for improving understanding of the heterogeneous presentations observed at the intersection of autism and gender diversity [20]. Future longitudinal studies incorporating standardized neurodevelopmental assessments, gender-related outcome measures, and long-term follow-up are needed to better characterize developmental trajectories, psychiatric outcomes, and support needs in this particularly vulnerable population.

## Conclusions

This case highlights the coexistence of ASD and persistent GI in an adolescent AFAB whose autism remained unrecognized until late adolescence. The presence of social camouflaging, a clinical presentation consistent with the female autism phenotype, and severe psychiatric comorbidity contributed to delayed diagnosis and increased clinical complexity. This report underscores the importance of comprehensive neurodevelopmental assessments in gender-diverse adolescents presenting with significant psychiatric symptoms, as early recognition of ASD may improve diagnostic accuracy, guide individualized interventions, and support better mental health outcomes.
